# Do local governments’ energy-saving target constraints inhibit financialization? Evidence from nonfinancial listed firms in China

**DOI:** 10.1371/journal.pone.0285342

**Published:** 2023-05-19

**Authors:** Gongjin Hu, Ying Yu, Qinwen Wang

**Affiliations:** 1 School of Accounting, Zhongnan University of Economics and Law, Wuhan, China; 2 School of Accounting, Anhui University of Finance and Economics, Bengbu, China; University of Baltistan, PAKISTAN

## Abstract

The negative impact of the financialization of non-financial firms cannot be ignored in China. However, existing studies neglect that the government environmental governance is an important influential factor in corporate investment decisions. Using a sample of China’s non-financial listed firms from 2007 to 2020, we examine the impact of local governments’ energy-saving target constraints on the financialization of local firms in terms of whether local governments set numerically specific energy-saving targets in the Government Work Reports. The main findings of this paper are as follows. First, local governments setting clear energy-saving targets inhibit local firms’ financialization and the result holds even after a series of robustness tests. Second, the negative association between local governments’ energy-saving target constraints and firm financialization is more pronounced among firms in eastern regions and green provinces. Third, the quality of firm information disclosure and local environmental public supervision enhance the inhibiting effect of local governments’ energy-saving target constraints on firm financialization. Fourth, local governments’ energy-saving target constraints restrain firm financialization by attracting more external analyst coverage and encouraging internal technological innovation. Moreover, this inhibiting effect can help reduce overinvestment and improve the total factor productivity of firms. Our study provides evidence supporting firm financialization studies from the novel perspective of government environmental governance.

## Introduction

**Fig 1:**
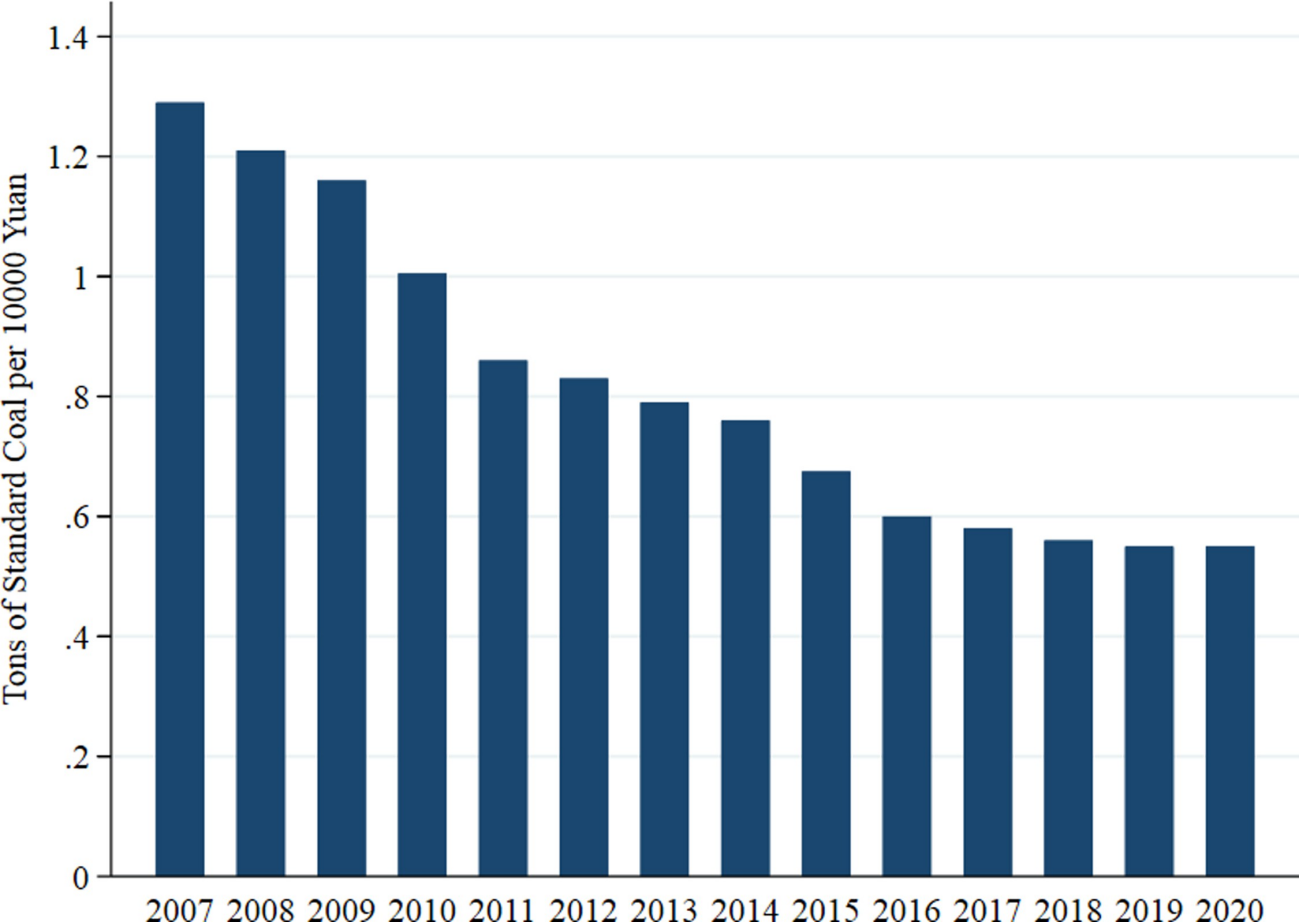
Energy consumption per 10,000 yuan of GDP from 2007 to 2020 in China.

Since the reform and opening-up, China’s economy has become a major driving force of global economic development with its continuously high growth rate. In recent years, along with the worldwide environmental deterioration and economic downturn, Chinese authorities have taken the initiative to adjust economic development goals and approaches in accordance with national conditions, i.e., shifting from high-speed growth to high-quality development, and the new development concept of ‘innovation, coordination, green, openness and sharing’ has produced initial results. Among them, the development of energy conservation and decarbonization is an important link. Many studies have found that energy price and efficiency have an important impact on firm performance, especially in terms of carbon taxes [1–5]. Compliance with the environmental governance goals set by the government, all parties concerned have made big efforts to lower energy consumption in practice for a long time and achieved remarkable results, as shown in Fig 1. To this end, we believe that the goal of government environmental governance plays a core guiding role in the process of high-quality development of economy and are bound to affect firm decision-making. However, few scholars have conducted research on firm investment and financing from the perspective of energy-saving target constraints set by the government.

At the same time, the development of real economy is the cornerstone of high-quality development. However, as the financial investments of Chinese firms increase, real investment is increasingly being crowded out. Firm financialization is the phenomenon of nonfinancial firms deviating from their main businesses and increasing their use of funds to purchase financial products and thus earn additional profits through nonrecurring operating channels [6]. The financialization of a large number of nonfinancial firms is not only detrimental to the development of their main businesses but also a factor leading to the formation of asset bubbles, which induce stock price crashes and introduce systemic financial risks. Therefore, an in-depth discussion of the motivations underlying firm financialization, an investigation of effective means to inhibit firms from financialization, and vigorous development of the real economy are conducive to the healthy and sustainable development of the national economy, which is of great significance in terms of promoting high-quality economic development.

Previous studies on the motivations underlying firm financialization show that the financial investments of firms are strongly influenced by their internal and external environments, and these studies mainly adopt the perspective of the individual economic environment. Investment substitution theory suggests that firms invest in financial assets because of the longer waiting period for investment returns in the context of real business and the possibility of lower returns [7]; moreover, the profit differential between real and financial business induces more firms to ignore their main businesses and invest large amounts of capital in financial assets with the hope of earning excess returns from arbitrage in financial markets [6]. However, according to the reservoir effect, firms make financial investments because financial assets can be quickly liquidated [8], which can ensure normal production and operation when there is a lack of funds for future development [9]. In addition, pressures to create shareholder value [10], technological changes [11], trust structures [12], growth in the cash holdings of nonfinancial firms [13], and government subsidies affect firms’ financial asset allocation [14]. Accordingly, the existing studies on the governance effects of financialization mainly focus on social trust and corporate social responsibility [15], stock market liberalization [16], the aspects of shareholder pay-outs [17], and market competition [18].

Obviously, the national governance environment, especially in terms of the impact of government environmental governance on the financialization behavior of firms, is neglected. Since local governments hold the power and responsibility to allocate local resources, which are bound to have an impact on firms’ investment behavior [19], it is important to pay attention to governments’ environmental resource governance systems when studying issues related to the financialization of firms [20]. Local governments’ energy-saving target constraints are an important part of China’s environmental system and an essential tool that helps achieve carbon emission peaks and carbon neutrality [21, 22]. Specifically, as assessments of local government performance increasingly focus on the efficient use of energy, energy-saving targets will constitute an increasingly important external environmental factor for firms, imposing a regulatory effect on their behavior, influencing their resource allocation and ultimately impacting their financial investment. On the one hand, compared with local governments that have not proposed clear energy-saving targets, local governments with clear energy-saving target constraints receive more attention from the public; moreover, local firms are likely to receive more attention from analysts and other outsiders, who effectively supervise firm behaviors and exert an impact on their financialization. On the other hand, the energy-saving target constraints of local governments reflect the importance of environmental resource utilization, and they directly impact firms in that they may encourage firms to make more effective real business investments for technological innovation, thereby reducing these firms’ financial investment and inhibiting the occurrence of financialization. Therefore, based on the characteristic evidence of local government’s energy-saving target constraints, we examine how the financialization of local firms is affected when local governments face energy-saving target constraints.

The contributions of this paper mainly correspond to the following three points. First, our paper explores the impact of local governments’ energy-saving target constraints on the financialization of nonfinancial firms, thus broadening the research perspective on the influencing factors and governance mechanism of financialization from the economic environment to the national governance environment. Second, this paper examines the specific mechanisms by which local governments with energy-saving target constraints guide firms to pay attention to the development of real business and prevent nonfinancial firms from engaging in financialization, and these mechanisms include attracting the attention of analysts and promoting the technological innovation of firms; in this way, we enrich the research on the impact of local governments’ energy-saving targets on firms’ behavior. Third, we construct a policy variable denoting local governments’ energy-saving target constraints by manually collecting the Government Work Reports of each prefecture-level city of China over the years examined, and we verify that local governments’ energy-saving target constraints can help achieve a win‒win situation of increased resource utilization and high-quality firm development; this result has positive policy implications for both governments and firms.

The rest of this paper is organized as follows. The second section presents the theoretical analysis and hypothesis proposition. The third section describes the research design. The fourth section provides an analysis of the main regression results. The fifth section presents robustness tests. The sixth section offers an additional analysis. Finally, the last section presents the conclusions and implications of our paper.

### Theoretical analysis and hypothesis proposition

It is commonly accepted that firms have motivations to make investments in financial assets, and these mainly consist of arbitrage motives and savings motives. The separation of ownership and operation rights tends to cause conflicts of interest between the managers and owners of a firm. This, however, verifies that firms are mainly motivated to invest in financial assets due to arbitrage. Specifically, in most cases, managers excessively pursue short-term goals that benefit themselves, while owners seek to maximize long-term benefits. As a result, managers may use their managerial power to make desirable decisions to achieve performance goals or satisfy their personal needs [23], earn contractual rewards [24], and generate surplus profits at the expense of owners [25]. Therefore, it is necessary to seek effective means to reduce these arbitrage incentives to reduce the financialization of firms. When the arbitrage motive underlying financial investment is reduced, the financial assets of firms are more available to play a role in sustaining their main businesses.

Since the Industrial Revolution, energy use has been thought of around the world as an important cornerstone of economic development [26]. Specifically, energy increasingly demonstrates a strong link to the overall development strength of countries, including the ability to reduce development costs and improve sustainable development capability [27], and play a significant role in the international policy landscape [28]. Consequently, the world’s leading countries often compete in terms of energy reserves and usage, aiming for a favorable position in terms of economic competition. However, the increasing prominence of global energy constraints and the resulting environmental problems cannot be ignored, which pose a major challenge to sustainable economic development. In this context, an increasing number of countries are making energy efficiency a key environmental governance and economic policy, such as the improvement of energy efficiency strategy within the framework of green economy concept in Russia [29], the launch of Energy Services Directives (ESD) in the European Union [30], and the implementation of energy saving policy of coal power generation in the United States [31].

**Fig 2:**
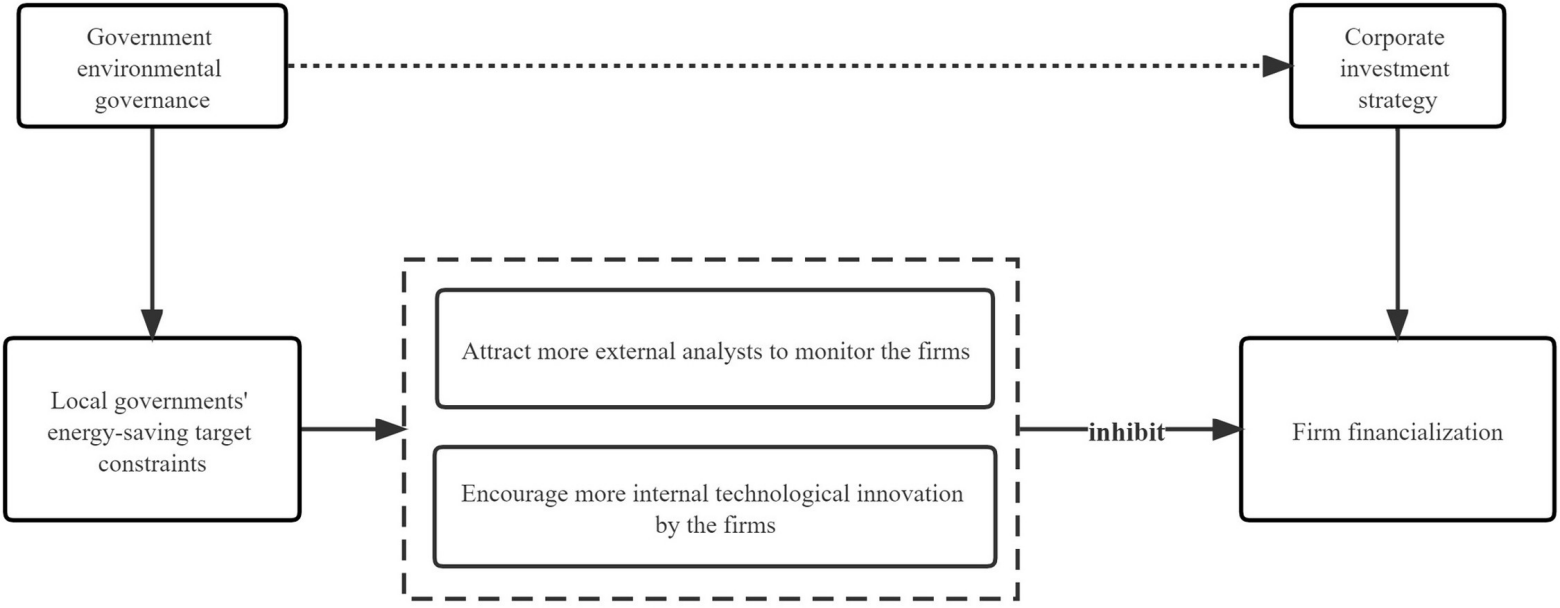
The framework of theoretical analysis.

As the world’s largest developing country, China’s energy-saving targets have been included in the performance appraisals of local governments, and one of the key forms is reflected in the annual Government Work Reports of local governments. In China, where the government owns and controls a large amount of economic and natural resources, the government can intervene directly in resource allocation, and local governments can also intervene in the behavior of local firms by providing financial subsidies or offering financing facilities. To ensure the achievement of energy-saving targets, local governments may intervene in local firms to induce them to shift their investment choices. Local governments’ energy-saving target constraints constitute a regulatory policy for firms. On the one hand, firms in areas with numerically specific local government energy-saving targets tend to receive more outside attention [32]; for example, they receive more analyst attention [33], which strengthens the monitoring of firm behavior and alleviates information asymmetries between internal and external investors [34]. This, in turn, has a supervisory effect on the self-interested behavior of corporate management, reduces excessive investment in financial assets in pursuit of short-term profits, and discourages the occurrence of firm financialization. On the other hand, local governments’ energy-saving target constraints can influence local firms to change their investment strategies [35], and promote technological innovation [36, 37], which can encourage firms to adapt to changes in the policy environment and focus on long-term business; in turn, this can reduce financial investment and mitigate the negative impact of the financialization of firms on the real economy. It can be seen that local governments’ energy-saving target constraints can play a supervisory and constraining role in encouraging firms to focus on real business development and discourage arbitrage, thereby reducing the financialization of firms. The framework of theoretical analysis in this paper is presented in Fig 2. Accordingly, we propose the following hypothesis:

**H1.** Local governments’ energy-saving target constraints can inhibit firm financialization.

## Research design

### Data sources and sample construction

Given that A-share listed firms play an important role in promoting China’s economic development and maintaining social stability, they are also key targets directly influenced by government policies. According to the existing literature, we select A-share listed firms in China as our research sample, and we begin by choosing the sample observation period of 2007 to 2020. We then exclude the following observations: financial firms, ST and PT firms, firms listed for less than one year, firms with asset-liability ratios greater than 1, and firms with missing data. Additionally, the prefecture-level city data involved in the study are mainly obtained from the Government Work Reports of all the prefecture-level cities, the China City Statistical Yearbook and the China Environment Statistical Yearbook, and the indicators involved, such as the marketization process indicator, are obtained from the China Marketization Index by Province Report (2018) compiled by Wang Xiaolu et al. The data for the latest year are calculated based on the average growth rate of the previous three years. The financial data are obtained from the China Stock Market & Accounting Research Database (CSMAR), the RESSET financial research database (RESSET) and the China Research Data Service Platform database (CNRDS). Moreover, all continuous variables are winsorized at the 1% and 99% quantiles to reduce the influence of outliers on the results, and 21,578 valid sample observations are finally obtained.

### Variable definition

#### Financialization

Firm financialization is usually defined as a greater preference for financial investment than for real investment; thus, according to the ‘activity-oriented perspective’ [38], it is appropriate to measure the financialization (FIN) of a firm based on the proportion of its investment income that comes from financial assets. Specifically, this is calculated as the sum of investment income, fair value change income and exchange gains minus investment income in associates and joint ventures, scaled by operating profits.

#### Energy-saving target constraints

We can obtain the variable of energy-saving target constraints (ESTCON) by manually collecting the Government Work Reports of all the prefecture-level cities of China from 2007 to 2020. If a specific numerical target for energy-saving assessments is disclosed in the focal work report, the variable is equal to 1; otherwise, it is equal to 0. For example, in the 2015 Beijing Municipal Government Work Report, an energy-saving target of ‘a 2% reduction in energy consumption per 10,000 yuan of GDP’ is clearly stated; in this case, we conclude that the Beijing Municipal Government had energy-saving target constraints in 2015. However, the ‘further reduction in energy consumption per unit of GDP’ proposed in the 2015 Shanghai Municipal Government Work Report does not explicitly include a value for the energy-saving target, so we conclude that the Shanghai Municipal Government was not constrained by specific energy-saving targets in 2015.

#### Control variables

We also include a set of control variables covering certain firm characteristics, corporate governance characteristics and regional characteristics; specifically, these are firm size (SIZE), asset-liability ratio (LEV), return on total assets (ROA), asset structure (FIX), executive compensation (PAY), board size (BSIZE), CEO duality (DUAL), ownership control (TOP2_10), marketisation process (MARKET), level of economic development (AGDP) and its squared value (AGDP^2^). The definitions of all the variables are shown in Table 1.

**Table 1:** Variable definition.

Variable type	Symbol	Name	Measure
**Dependent variable**	FIN	Degree of non-financial firm financialization	The sum of investment income, fair value change income and exchange gains minus investment income in associates and joint ventures, scaled by operating profits.
**Independent variable**	ESTCON	Energy-saving target constraints	Dummy variable that equals 1 if the local Government Work Report for a particular year announces specific figures for energy-saving targets, and 0 otherwise.
**Control variable**	SIZE	Firm size	Natural logarithm of total assets.
	LEV	Asset-liability ratio	Total liabilities divided by total assets.
	ROA	Return on total assets	Net profit divided by total assets.
	FIX	Asset structure	Fixed assets divided by total assets.
	PAY	Executive compensation	The sum of the top three executive compensation divided by total assets.
	BSIZE	Board size	Natural logarithm of the number of board members.
	DUAL	CEO duality	Dummy variable that equals 1 if CEO and the chairman are the same person, and 0 otherwise.
	TOP2_10	Ownership control	The sum of the shareholdings of the second largest shareholder to the tenth largest shareholder of the firm.
	MARKET	Marketisation process	The overall score for each region’s marketization process provided by the report.
	AGDP	Level of economic development (a)	Natural logarithm of GDP per capita.
	AGDP^2^	Level of economic development (b)	Square of the natural logarithm of GDP per capita.

### Regression model

To better reflect the research results of our paper intuitively, we choose to use stata16 software to perform OLS estimation on the following model to verify hypothesis 1:

$$FIN_{it}=\alpha_{0}+\alpha_{1}ESTCON_{it}+\alpha_{2}CONTROL_{it}+YEAR+IND+\varepsilon_{it}$$ (1)

In model (1), *ε_it_* is the error term, and *α*_1_ is the focus of our paper, i.e., the regression coefficient of *ESTCON*_it_, which mainly measures the effect of local governments’ energy-saving target constraints on the financialization of firms. If *α*_1_ is significantly negative, this indicates that local governments’ energy-saving target constraints reduce the financial investment of firms, which can inhibit the trend of ‘moving away from the real economy to the virtual economy’ exhibited by nonfinancial firms. Both year and industry effects are controlled in subsequent regressions.

## Analysis of main regression results

### Descriptive statistics

Table 2 reports the descriptive statistics of the main variables. According to this table, the mean value of FIN is 0.053, the median value is 0, the minimum value is -0.487, the maximum value is 1.835, and the overall standard deviation is 0.250, which shows that there are significant individual differences in the degree of firm financialization and that these fluctuations are large. The overall standard deviation of ESTCON is 0.463, indicating that there are obvious differences across the energy-saving target constraints of different regions. The distribution of the control variables is generally consistent with prior literature.

**Table 2:** Descriptive statistics.

Variable	N	Mean	Overall Sd	Between Sd	Within Sd	Min	p25	p50	p75	Max
**FIN**	21578	0.053	0.250	0.091	0.228	-0.487	0	0	0.002	1.835
**ESTCON**	21578	0.312	0.463	0.318	0.346	0	0	0	1	1
**SIZE**	21578	22.164	1.192	1.030	0.589	19.926	21.303	22.028	22.882	25.749
**LEV**	21578	0.439	0.200	0.178	0.096	0.06	0.283	0.436	0.591	0.868
**ROA**	21578	0.043	0.052	0.038	0.038	-0.161	0.016	0.039	0.068	0.196
**FIX**	21578	0.231	0.167	0.146	0.075	0.002	0.100	0.200	0.329	0.727
**PAY**	21578	0.001	0.001	0.001	0.000	0	0	0	0.001	0.003
**BSIZE**	21578	2.151	0.194	0.173	0.097	1.609	2.079	2.197	2.197	2.708
**DUAL**	21578	0.762	0.426	0.383	0.258	0	1	1	1	1
**TOP2_10**	21578	22.368	12.922	11.841	6.916	2.052	11.728	21.188	31.451	54.326
**MARKET**	21578	8.213	2.030	1.916	0.867	-1.420	6.850	8.300	9.870	12.000
**AGDP**	21578	11.269	0.604	0.518	0.317	8.615	10.918	11.333	11.727	13.056
**AGDP** ^**2**^	21578	127.348	13.403	11.555	7.031	74.225	119.209	128.443	137.515	170.451

### Stationary test

To ensure the validity of the subsequent regression analysis, the Fisher-ADF stationary test and Fisher-PP stationary test have been conducted for the key variables in this paper. From the results of the test in Table 3, it can be seen that the p-values are significantly less than 0.01, and the original hypothesis that the variables have unit roots is strongly rejected, so the key variables all pass the stationary test and can be analyzed by regression.

**Table 3:** Stationary test.

Variable	Fisher-ADF		Fisher-PP		Conclusion
	Statistics	p-values	Statistics	p-values	
FIN	129.444	0.000	281.669	0.000	Stationary
ESTCON	109.087	0.000	103.702	0.000	Stationary

### Main regression results

Table 4 presents the influence of local governments’ energy-saving target constraints on firm financialization, i.e., the main regression results of model (1). In column (1), the regression coefficient of ESTCON without the inclusion of the control variables is -0.013 and significantly negative at the 1% level; moreover, when all the control variables are added, as shown in Column (2), the ESTCON coefficient is -0.014 and remains significant at the 1% level. Our baseline regression results document that when a local government is constrained by specific energy-saving targets, the firms in the local area are less financialized; that is, local governments’ energy-saving target constraints can help reduce the financial investment of local firms, inhibit the financialization and prevent firms from ‘moving away from the real economy to the virtual economy’. In summary, hypothesis 1 is true.

**Table 4:** Main results.

	(1)	(2)
	FIN	FIN
ESTCON	-0.013***	-0.014***
	**(-2.909)**	**(-3.163)**
SIZE		0.002
		(1.001)
LEV		-0.010
		(-0.767)
ROA		-0.091***
		(-2.954)
FIX		-0.117***
		(-8.541)
PAY		-1.576
		(-0.478)
BSIZE		-0.015
		(-1.531)
DUAL		0.010***
		(2.771)
TOP2_10		-0.001***
		(-9.155)
MARKET		0.002
		(1.566)
AGDP		0.167**
		(2.109)
AGDP^2^		-0.008**
		(-2.190)
REGION	YES	YES
YEAR	YES	YES
IND	YES	YES
_cons	0.085***	-0.802*
	(4.319)	(-1.812)
N	21578	21578
Adj-R^2^	0.052	0.059

**NOTE:** ***, **, and * denote significance at the 1%, 5%, and 10% levels, respectively. The t-values based on heteroskedasticity robust standard errors are in parentheses, same as below.

## Robustness test

### Alternative definition of financialization

According to prior studies, financialization can also be measured based on the amount of financial assets invested by a firm. Therefore, following Demir (2009) [6], we use the ratio of financial assets to total assets to measure the degree of firm financialization and repeat the main test. More specifically, this alternative measure is calculated as the sum of trading financial assets, derivative instruments, available-for-sale investments, held-to-maturity investments, long-term equity investments, and investment property, scaled by total assets, with a larger indicator indicating a higher degree of firm financialization. As shown in S1 Table, regardless of whether the control variables are added, the coefficient of ESTCON is negative and significant at the 5% level and 1% level, respectively, indicating that our main findings continue to hold.

### Delete real estate industry samples

As the real estate industry is recognized as a ‘profiteering’ industry and characteristics related to the financial attributes of commodity housing are showing an increasing trend in China, real estate firms can be defined as financial firms in a certain sense. Therefore, we exclude any real estate industry data from the original sample and conduct the regression test again; moreover, the results in S2 Table show that the ESTCON coefficients are -0.013 and -0.014 with and without the inclusion of control variables, respectively, and both are significant at the 1% level. Thus, our main results remain unchanged.

### Endogeneity test: Propensity score matching and entropy balancing

Considering that our results may stem from differences among firms themselves rather than being influenced by whether the local government has energy-saving target constraints, we adopt the propensity score matching (PSM) method to alleviate this endogeneity problem. We use the nearest-neighbour matching method to match firms under local governments with energy-saving target constraints with firms under local governments that do not have energy-saving target constraints; subsequently, we regress again, and the results are shown in S3 Table Panel A and Panel B. In Panel A, all variables are not significant at the 5% level after matching, which indicates that the means of these variables are not significantly different after pairing and that the matching is valid. In the regression results shown in Panel B, the ESTCON coefficient without the inclusion of the control variables is -0.019 and significant at the 5% level, and after adding control variables, we find that this coefficient is -0.020 and significant at the 1% level, which shows that our hypothesis still holds.

In addition, we use the entropy balancing method for the matching process, and the specific regression results are listed in S3 Table Panel C. The regression coefficient of ESTCON is -0.015 and significant at the 1% level irrespective of whether control variables are included, further validating our findings.

### Energy-saving target constraints intensity analysis

In addition, to further verify whether there are differences in the impact of the constraint levels within these samples with energy-saving target constraints, we measure the intensity of the energy-saving target constraints based on the proportion of the frequency of words related to energy-saving target and environmental protection in the Government Work Reports to the number of words in the full Government Work Reports, and run the regression again, where the relevant word frequencies include low carbon, environmental protection, air, green, PM2.5, COD, CO2, PM10, ecology, emissions, emission reduction, pollution, environmental protection, SO2, energy consumption, etc. In the specific regression process, the samples are divided into high and low groups according to the mean value of constraint intensity, and the related results are presented in S4 Table. According to the regression results, it can be found that the regression coefficient of energy-saving target constraints intensity on firm financialization is -9.686 in the high constraint sample group and significant at the 5% level, while it is 4.392 in the low constraint sample group and does not pass the significance test. The conclusions suggest that the higher the intensity of the energy-saving target constraints, the more conducive it is to curb the firm financialization, further corroborating the findings of this paper.

## Additional analysis

### Cross-sectional analysis

#### Regional heterogeneity

Due to the vast geographical distribution of China, local government policies and regional development levels vary across different regions. Compared with the central and western regions, the eastern regions have more rapid economic development, account for a greater proportion of the use of various resources, and a better legal environment; therefore, local governments in the eastern regions may be more stringent in their implementation of energy-saving targets. Based on the above, we divide the sample into an eastern regions sample and a central and western regions sample for another multiple regression, and the regression results are shown in columns (1) and (2) of Table 5. As shown in these columns, the ESTCON regression coefficient of the eastern regions sample in column (1) is -0.017 and significant at the 1% level, while the regression coefficient of the central and western regions sample in column (2) is -0.008 and does not pass the significance test. The p value of the coefficient difference test between the groups is 0, indicating that the inhibitory effect of local governments’ energy-saving target constraints on firm financialization differs significantly at the 1% level depending on the nature of the region, with the effect being more pronounced among eastern regions firms.

**Table 5:** The results of cross-sectional analysis.

	(1)	(2)	(3)	(4)
	Eastern regions	Central and western regions	Green provinces	Polluting provinces
ESTCON	-0.017***	**-0.008**	-0.030***	**-0.006**
	**(-2.995)**	**(-0.943)**	**(-3.879)**	**(-0.917)**
SIZE	0.000	0.009*	0.004	0.001
	(0.129)	(1.921)	(0.967)	(0.374)
LEV	0.004	-0.047*	-0.007	-0.006
	(0.233)	(-1.942)	(-0.310)	(-0.355)
ROA	-0.114***	-0.038	-0.132**	-0.042
	(-3.164)	(-0.629)	(-2.306)	(-1.178)
FIX	-0.149***	-0.071***	-0.135***	-0.094***
	(-8.759)	(-2.961)	(-5.532)	(-5.727)
PAY	-5.670*	10.388	-0.593	-3.783
	(-1.645)	(1.298)	(-0.089)	(-1.023)
BSIZE	-0.008	-0.026	-0.037**	-0.001
	(-0.701)	(-1.466)	(-2.083)	(-0.043)
DUAL	0.013***	-0.001	0.017**	0.007
	(3.353)	(-0.104)	(2.355)	(1.611)
TOP2_10	-0.001***	-0.002***	-0.001***	-0.001***
	(-7.070)	(-6.024)	(-5.189)	(-7.373)
MARKET	0.004**	0.000	-0.000	0.004***
	(2.000)	(0.067)	(-0.063)	(2.635)
AGDP	0.103	0.052	0.302*	0.085
	(0.758)	(0.307)	(1.780)	(0.947)
AGDP^2^	-0.005	-0.002	-0.014*	-0.004
	(-0.874)	(-0.248)	(-1.793)	(-1.051)
YEAR	YES	YES	YES	YES
IND	YES	YES	YES	YES
_cons	-0.419	-0.331	-1.448	-0.410
	(-0.538)	(-0.364)	(-1.557)	(-0.815)
N	14642	6936	7795	13783
Adj-R^2^	0.069	0.049	0.072	0.054
Diff(P-value)	0.000		0.000	

#### Environmental regulatory heterogeneity

Similarly, the environmental regulatory requirements of different regions may cause local governments’ energy-saving target constraints to have different effects on firm financialization. Therefore, we divide the sample into a group of firms in green provinces and a group in polluting provinces to run multiple regressions. The division principle is based on the industrial SO2 emissions of 31 provinces and regions, which are standardized and then divided into two subsamples of listed firms according to their averages, and the results are presented in columns (3) and (4) of Table 5. As reported, the regression coefficient of ESTCON for the green province sample group in column (3) is -0.030 and is significant at the 1% level, while the regression coefficient for the polluted province sample group in column (4) is -0.006 and does not pass the significance test. The p value of the coefficient difference test between groups is 0, which indicates that the inhibitory effect of local governments’ energy-saving target constraints on firm financialization is significantly different at the 1% level depending on the nature of the applicable environmental regulation, with the relevant effect being more obvious among firms in green provinces. The possible reason for this is that the green provinces themselves have stronger external environmental enforcement and the firms themselves are more environmentally conscious and respond more effectively to the environmental targets set by the government, so the impact of the polluting provinces is relatively less pronounced than in the green provinces.

### Moderating effect

#### Quality of information disclosure

In general, the higher the quality of a firm’s information disclosure is, the more information asymmetry is reduced within and outside the firm, which in turn is conducive to reducing excessive financial investment by firm executives in pursuit of short-term interests. The quality of information disclosure is measured based on the information disclosure quality evaluations of the Shenzhen Stock Exchange and the Shanghai Stock Exchange, which are divided into four grades: ‘excellent’, ‘good’, ‘qualified’ and ‘unqualified’ or A, B, C and D. Thus, we construct the disclosure quality variable (DISC), assign the values of ‘excellent’ (A) as ‘4’, ‘good’ (B) as ‘3’, ‘qualified’ (C) as ‘2’, and ‘unqualified’ (D) as ‘1’, and design the following model for regression.


$$FIN_{it}=\alpha_{0}+\alpha_{1}ESTCON_{it}+\alpha_{2}\;DISC{\;}_{it}+\alpha_{3}ESTCON_{it}\times DISC_{it}+\alpha_{4}\;CONTROL{\;}_{it}+YEAR\;+\;IND+\varepsilon_{it}$$ (2)

The specific regression results are shown in columns (1) and (2) of Table 6. The coefficient of the interaction term, ESTCON×DISC, is significantly negative at the 10% level regardless of whether the control variables are included, suggesting that an increase in the quality of information disclosure enhances the restraining effect of local governments’ energy-saving target constraints on firm financialization.

**Table 6:** The results of moderating effect.

	(1)	(2)	(3)	(4)
	FIN	FIN	FIN	FIN
ESTCON	0.036	0.034	-0.005	-0.007
	(1.276)	(1.223)	(-0.747)	(-0.987)
DISC	-0.010**	-0.009**		
	(-2.560)	(-2.268)		
ESTCON×DISC	-0.014*	-0.014*		
	**(-1.721)**	**(-1.662)**		
PENALTY			-0.000	-0.000
			(-1.618)	(-1.476)
ESTCON×PENALTY			-0.000*	-0.000*
			**(-1.802)**	**(-1.781)**
SIZE		0.002		0.002
		(0.632)		(1.024)
LEV		-0.010		-0.010
		(-0.570)		(-0.763)
ROA		-0.048		-0.089***
		(-1.215)		(-2.887)
FIX		-0.121***		-0.116***
		(-6.369)		(-8.472)
PAY		-4.509		-1.467
		(-1.094)		(-0.445)
BSIZE		-0.025**		-0.015
		(-1.970)		(-1.508)
DUAL		0.012**		0.010***
		(2.403)		(2.744)
TOP2_10		-0.001***		-0.001***
		(-6.452)		(-9.126)
MARKET		0.001		0.002*
		(0.845)		(1.769)
AGDP		0.158**		0.154*
		(1.966)		(1.927)
AGDP^2^		-0.008**		-0.007**
		(-2.103)		(-2.000)
YEAR	YES	YES	YES	YES
IND	YES	YES	YES	YES
_cons	0.119***	-0.651	0.082***	-0.733*
	(3.689)	(-1.438)	(4.176)	(-1.650)
N	11682	11682	21578	21578
Adj-R^2^	0.056	0.064	0.052	0.060

#### Environmental public supervision

In terms of the external information environment, the stronger the local environmental public supervision is, the more cautious firms are when using resources. Therefore, we select the number of administrative penalty cases to measure the intensity of environmental public supervision in the area where each firm is located, denote this variable as PENALTY, and use the following model for regression.


$$\;FIN_{it}=\alpha_{0}+\alpha_{1}\;ESTCON{\;}_{it}+\alpha_{2}\;PENALTY_{it}+\alpha_{3}\;ESTCON_{it}\times \;PENALTY_{it}+\alpha_{4}\;CONTROL{\;}_{it}+YEAR\;+IND+\;\varepsilon_{it}$$ (3)

The regression results are reported in columns (3) and (4) of Table 6, and the coefficients of ESTCON×PENALTY are significantly negative at the 10% level regardless of whether control variables are added, which demonstrates that local environmental public supervision promotes the governance effect of local governments’ energy-saving target constraints on firm financialization.

### Mediating effect

#### Analyst coverage

Based on the above discussion, regions with explicit energy-saving targets at the local government level tend to receive more attention from external sources than regions without clear energy-saving targets at this level; thus, such targets function as an effective institutional constraint. For firms specifically, this external attention may attract more analyst coverage, which in turn enhances the external supervision power of firms, makes them more focused on their main businesses and long-term development and causes them to be less likely to invest in financial assets; this ultimately discourages financialization. Therefore, we argue that analyst coverage is a possible mechanism by which local governments’ energy-saving target constraints inhibit firm financialization, and we use the following models to verify this hypothesis.


$$ATTEN_{it}=\alpha_{0}+\alpha_{1}ESTCON_{it}+\alpha_{2}CONTROL_{it}+YEAR+IND+\varepsilon_{it}$$ (4)

$$FIN_{it}=\alpha_{0}+\alpha_{1}\;ESTCON_{it}+\alpha_{2}\;ATTEN_{it}+\alpha_{3}\;CONTROL_{it}+YEAR+IND+\varepsilon_{it}$$ (5)

In these models, ATTEN is the possible mediating variable, and the variable value is the natural logarithm of the number of analysts following each firm. The regression results are shown in Table 7. Column (2) shows the regression results of model (4) with a regression coefficient of ESTCON that is equal to 0.359 and significantly at the 1% level, which indicates that local governments’ energy-saving target constraints can cause local firms to attract more analysts’ attention. The regression results of model (5) are reported in column (3). The regression coefficient of ESTCON is -0.014 and significant at the 1% level, and the regression coefficient of ATTEN is -0.002 and significant at the 1% level. Combining the regression results of model (1), we use the three significance tests provided by the Sgmediation command, i.e., Sobel, Goodman1, and Goodman2 tests, to perform a mediating effect test, and these results all pass the significance test. Our results document that analyst coverage plays a partially mediating role in the impact of local governments’ energy-saving target constraints on firm financialization. That is, attracting analysts’ attention is one of the channels through which local governments’ energy-saving target reduce the firm financialization.

**Table 7:** The results of mediating effect.

	(1)	(2)	(3)	(4)	(5)
	FIN	ATTEN	FIN	TECH	FIN
ESTCON	-0.014***	0.359***	-0.014***	0.176***	-0.014***
	**(-3.164)**	**(2.783)**	**(-3.024)**	**(4.077)**	**(-3.096)**
ATTEN			-0.002***		
			**(-10.988)**		
TECH					-0.002**
					**(-2.426)**
SIZE	0.002	3.781***	0.009***	0.066***	0.002
	(0.963)	(45.791)	(3.767)	(3.033)	(1.011)
LEV	-0.009	-2.279***	-0.014	-2.972***	-0.014
	(-0.721)	(-6.474)	(-1.054)	(-23.004)	(-1.091)
ROA	-0.090***	52.474***	0.009	-1.776***	-0.093***
	(-2.929)	(38.002)	(0.282)	(-3.674)	(-3.020)
FIX	-0.117***	0.670	-0.116***	-0.283**	-0.118***
	(-8.515)	(1.599)	(-8.445)	(-2.340)	(-8.553)
PAY	-1.595	884.207***	0.073	228.929***	-1.217
	(-0.484)	(7.585)	(0.022)	(5.220)	(-0.368)
BSIZE	-0.015	-0.594*	-0.016	-0.147	-0.015
	(-1.483)	(-1.947)	(-1.599)	(-1.484)	(-1.508)
DUAL	0.010***	-0.910***	0.008**	-0.242***	0.010***
	(2.756)	(-6.846)	(2.301)	(-5.354)	(2.652)
TOP2_10	-0.001***	0.056***	-0.001***	0.009***	-0.001***
	(-9.163)	(12.972)	(-8.395)	(6.614)	(-9.065)
MARKET	0.001	0.125***	0.001	0.034***	0.001
	(1.105)	(3.627)	(1.315)	(2.988)	(1.157)
AGDP	0.164**	-0.911	0.163**	0.184	0.165**
	(2.077)	(-0.372)	(2.054)	(0.272)	(2.082)
AGDP^2^	-0.008**	0.037	-0.008**	-0.001	-0.008**
	(-2.167)	(0.338)	(-2.146)	(-0.038)	(-2.169)
YEAR	YES	YES	YES	YES	YES
IND	YES	YES	YES	YES	YES
_cons	-0.775*	-71.704***	-0.911**	-1.762	-0.778*
	(-1.760)	(-5.245)	(-2.059)	(-0.474)	(-1.768)
N	21578	21578	21578	21578	21578
Adj-R^2^	0.059	0.313	0.063	0.474	0.060
Sobel test		-0.0012*** (Z = -4.209)		-0.0004** (Z = -2.064)	
Goodman test 1		-0.0012*** (Z = -4.191)		-0.0004** (Z = -2.041)	
Goodman test 2		-0.0012*** (Z = -4.227)		-0.0004** (Z = -2.088)	
Mediating effect coefficient		-0.0012*** (Z = -4.209)		-0.0004** (Z = -2.064)	
Direct effect coefficient		-0.0117*** (Z = -2.951)		-0.0127*** (Z = -3.158)	
Total effect coefficient		-0.0130*** (Z = -3.255)		-0.0130*** (Z = -3.254)	

#### Technology innovation

In addition, to achieve energy-saving targets, local governments may impose energy-saving requirements on local firms, which may encourage firms to carry out technological innovation, invest more in R&D projects and reduce their investment in financial assets to meet these requirements. Thus, we suggest that firms’ technological innovation may also be a mediating variable in the impact of local governments’ energy-saving target constraints on firm financialization. Similarly, we estimate the following regression model for verification.


$$TECH{\;}_{it}=\alpha_{0}+\alpha_{1}\;ESTCON_{it}+\alpha_{2}\;CONTROL_{it}+YEAR+IND+\varepsilon_{it}$$ (6)

$$FIN_{it}=\alpha_{0}+\alpha_{1}\;ESTCON_{it}+\alpha_{2}\;TECH_{it}+\alpha_{3}\;CONTROL_{it}+YEAR+IND+\varepsilon_{it}$$ (7)

In these models, TECH is the possible mediating variable, the value of which is the ratio of a firm’s R&D investment to its operating profit, and the regression results are presented in Table 7. Column (4) shows the regression results of model (6) with a regression coefficient of ESTCON that is equal to 0.176 and significantly at the 1% level, which indicates that local governments’ energy-saving target constraints can encourage more technological innovation by local firms. The regression results of model (7) are shown in column (5), and the regression coefficient of ESTCON is -0.014 and significant at the 1% level, while the regression coefficient of TECH is -0.002 and significant at the 5% level. For TECH, we also use the three significance tests provided by the Sgmediation command, i.e., Sobel, Goodman1, and Goodman2 tests, to perform a mediating effect test, combining the regression results of model (1), and the results all pass the significance test. These results verify that technological innovation plays a partial mediating role, i.e., local governments’ energy-saving target constraints promote the technological innovation of firms and thus discourage their financialization, and promoting technological innovation is also one of the channels through which local governments’ energy-saving target reduce the firm financialization.

### Economic consequences

#### Investment level

Since firms’ overinvestment in financial assets limits the development of their main business investment to a certain extent and local governments’ energy-saving target constraints are conducive to inhibiting financial investment, these targets may reduce firms’ overinvestment behaviour and enhance their level of investment. We use the following regression models to test this hypothesis.


$$NI_{it}=\alpha_{0}+\alpha_{1}\;GROWTH_{it-1}+\alpha_{2}LEV_{it-1}+\alpha_{3}\;CASH_{it-1}+\alpha_{4}AGE_{it-1}+\alpha_{5}SIZE_{it-1}+\;\alpha_{6}\;RET_{it-1}\;+\alpha_{7}\;NI_{it-1}\;+\;YEAR\;+IND+\;\varepsilon_{it}$$ (8)

$$INV_{it}=\alpha_{0}+\alpha_{1}\;ESTCON_{it}\times FIN_{it}+\alpha_{2}\;FIN_{it}+\alpha_{3}\;ESTCON_{it}+\alpha_{4}\;CONTROL_{it}+YEAR+IND+\varepsilon_{it}$$ (9)

To measure whether a firm is overinvested, we follow Richardson (2006) [39] to use model (8) to employ yearly OLS regressions to obtain the residuals of the model, that is, the variable of overinvestment (INV). Specifically, in model (8), *NI*_*it*_ is the actual new investment expenditure of firm i in year t, *GROWTH*_*it*-1_ is the growth rate of its main business revenue, *LEV*_*it*-1_ is the firm’s asset-liability ratio, *CASH*_*it*-1_ is the cash flow position, *AGE*_*it-*1_ is the firm’s age, *SIZE*_*it*-1_ is the firm’s asset size, and *RET_it_*_-1_ is the firm’s stock return. Overinvestment (INV) is a dummy variable that equals 1 if the residual is positive and 0 otherwise.

The regression results of model (9) are presented in columns (1) and (2) of Table 8. The coefficient of the interaction term ESTCON×FIN is significantly negative at the 5% level regardless of whether the control variables are added, suggesting that the inhibiting effect of local governments’ energy-saving target constraints on firm financialization is conducive to reducing overinvestment and improving the overall level of firm investment.

**Table 8:** The analysis of economic consequences.

	(1)	(2)	(3)	(4)
	INV	INV	TFP	TFP
FIN	0.027	0.025	-0.086**	-0.138***
	(1.536)	(1.423)	(-1.996)	(-5.643)
ESTCON	-0.011	-0.013	0.115***	0.006
	(-1.271)	(-1.512)	(6.163)	(0.674)
ESTCON×FIN	-0.062**	-0.062**	**0.047**	0.081**
	**(-2.289)**	**(-2.295)**	**(0.719)**	**(2.253)**
SIZE		0.028***		0.845***
		(6.026)		(161.518)
LEV		0.154***		0.841***
		(6.626)		(28.346)
ROA		0.324***		2.970***
		(4.474)		(31.998)
FIX		-0.162***		-0.151***
		(-6.052)		(-4.390)
PAY		-12.146*		65.643***
		(-1.698)		(7.721)
BSIZE		-0.024		0.004
		(-1.276)		(0.176)
DUAL		-0.007		0.050***
		(-0.833)		(5.733)
TOP2_10		0.001***		-0.001***
		(4.551)		(-2.924)
MARKET		0.003		0.032***
		(1.318)		(11.893)
AGDP		0.004		0.550***
		(0.027)		(3.274)
AGDP^2^		-0.001		-0.024***
		(-0.108)		(-3.226)
YEAR	YES	YES	YES	YES
IND	YES	YES	YES	YES
_cons	0.401***	-0.170	-0.085	-21.985***
	(8.213)	(-0.205)	(-0.864)	(-23.431)
N	21578	21578	21578	21578
Adj-R^2^	0.010	0.022	0.004	0.752

#### Total factor productivity

As discussed above, local governments’ energy-saving target constraints may encourage firms to carry out relevant technological innovation and reduce their financial investment, which may improve their total factor productivity to a certain extent and thus promote high-quality economic development. Similarly, we estimate the following regression model for the test.


$$TFP_{it}=\alpha_{0}+\alpha_{1}\;ESTCON\times FIN_{it}+\alpha_{2}\;FIN_{it}+\alpha_{3}\;ESTCON_{it}+\alpha_{4}\;CONTROL_{it}+YEAR+IND+\varepsilon_{it}$$ (10)

In model (10), TFP refers to the total factor productivity of the focal firm. The substantial body of literature examining TFP is premised on the semiparametric method proposed by Olley and Pakes (1996) [40] and Levinsohn and Petrin (2003) [41], i.e., the OP method and the LP method. However, considering that the OP method requires that real firm investment be greater than 0, which may lead to the loss of some of our samples in the estimation process, we adopt the LP method to measure the total factor productivity variable. The regression results of model (10) are shown in columns (3) and (4) of Table 8. When no control variables are added, the coefficient of the interaction term ESTCON×FIN is 0.047, and it does not pass the significance test; however, after including the control variables, we find that the coefficient of ESTCON×FIN is 0.081 and significant at the 5% level, which implies that to some extent, the inhibiting effect of local governments’ energy-saving target constraints on the financialization of nonfinancial firms is conducive to improving the total factor productivity of firms and enhancing their overall development quality.

## Conclusions and policy recommendations

Environmental governance is an essential part of high-quality economic development, and the environmental governance targets set by the government play a core guiding role in it. As the main body of real economic development, the decision-making behavior of firms is bound to be influenced by the government environmental governance. However, as the financial investments of Chinese nonfinancial firms increase, real investment is increasingly being crowded out, which is to the detriment of main businesses and may induce systemic financial risks. Whether the goals of government environmental governance have a governance effect on the firm financialization is an interesting and important question, but there is no literature that specifically studies this. Therefore, we choose nonfinancial A-share firms in China from 2007 to 2020 as our research sample and investigate the impact of local governments’ energy-saving target constraints on the financialization of firms.

The main results show the following: (1) Local governments’ energy-saving target constraints can inhibit local firms’ financialization, which validates our hypothesis. (2) Local governments’ energy-saving target constraints have a more significant inhibitory effect on firm financialization in the eastern regions and green provinces than in the central and western regions and polluting provinces. (3) The higher the quality of firm information disclosure and stronger environmental public supervision are, the more the impact of local governments’ energy-saving target constraints on financialization is strengthened. (4) Local governments’ energy-saving target constraints reduce firm financialization by attracting more attention from external analysts and promoting more internal technological innovation, proving that attracting analysts’ attention and promoting technological innovation are possible channels of reducing the firm financialization under energy-saving target. (5) Local governments’ energy-saving target constraints restrict financialization, which is conducive to reducing firms’ overinvestment and increasing total factor productivity, that is, promoting high-quality economic development.

Our findings have clear policy recommendations for how to make concerted efforts to guide firms to focus on the development of real economy. On the one hand, local governments should continue to implement the concept of green, sustainable and healthy development, clarify environmental governance goals, improve the relevant legal systems, and introduce relevant preferential and incentive policies that encourage firms to focus on energy-saving targets and green development models for real business. On the other hand, firms should establish a long-term vision of development, increase relevant technological innovation, continuously improve their industry competitiveness, reduce their motivation to pursue short-term profits through excessive allocation of financial assets, and achieve long-term healthy development. Ultimately, this will lead to a win‒win situation in terms of both the efficient use of environmental resources and healthy economic development.

## Limitations and outlook

Our paper mainly has the following limitations. The targets of government environmental governance are diverse, and we choose the local governments’ energy-saving target constraints as a proxy variable that cannot fully represent the overall environmental governance targets. Meanwhile, given the limitations of the Government Work Report data itself, we use a dummy variable for the local governments’ energy-saving target constraints variable, which to some extent cannot capture the impact of the intensity of energy-saving target governance on firm financialization. Therefore, future studies should find more overall representative proxy variables for environmental governance goals by further exploring government information disclosure. In addition, the environmental governance goals can be measured specifically by combining the methods of textual analysis to better represent the strength of environmental governance targets.

## Supporting information

S1 Data (DTA)

S1 Table Alternative definition of financialization. (DOCX)

S2 Table Delete the samples of real estate sector. (DOCX)

S3 Table Propensity score matching and entropy balancing. (DOCX)

S4 Table Energy-saving target constraints intensity analysis. (DOCX)
