# Supplementary material for: Do local governments’ energy-saving target constraints inhibit financialization? Evidence from nonfinancial listed firms in China
Source: PLoS One. 2023 May 19;18(5):e0285342. doi: 10.1371/journal.pone.0285342 (PMC10198514; doi:10.1371/journal.pone.0285342)
Supplement: S1 Table — (DOCX) [file pone.0285342.s002.docx]

**S1 Table**. **Alternative definition of financialization**

|  | **(1)** | **(2)** |
| --- | --- | --- |
|  | **FIN** | **FIN** |
| ESTCON | **-0.001**** | **-0.001***** |
|  | **(-2.095)** | **(-2.653)** |
| SIZE |  | -0.000* |
|  |  | (-1.934) |
| LEV |  | -0.003** |
|  |  | (-2.279) |
| ROA |  | 0.004 |
|  |  | (1.132) |
| FIX |  | -0.015*** |
|  |  | (-11.200) |
| PAY |  | -0.085 |
|  |  | (-0.232) |
| BSIZE |  | -0.002** |
|  |  | (-2.007) |
| DUAL |  | 0.001* |
|  |  | (1.757) |
| TOP2_10 |  | -0.000*** |
|  |  | (-10.771) |
| MARKET |  | 0.000*** |
|  |  | (3.993) |
| AGDP |  | -0.004 |
|  |  | (-0.595) |
| AGDP^2^ |  | 0.000 |
|  |  | (0.575) |
| YEAR | YES | YES |
| IND | YES | YES |
| _cons | -0.001 | 0.038 |
|  | (-0.322) | (1.041) |
| N | 21578 | 21578 |
| Adj-R^2^ | 0.204 | 0.215 |
