# Supplementary material for: Do local governments’ energy-saving target constraints inhibit financialization? Evidence from nonfinancial listed firms in China
Source: PLoS One. 2023 May 19;18(5):e0285342. doi: 10.1371/journal.pone.0285342 (PMC10198514; doi:10.1371/journal.pone.0285342)
Supplement: S2 Table — (DOCX) [file pone.0285342.s003.docx]

**S2 Table**. **Delete the samples of real estate sector**

|  | **(1)** | **(2)** |
| --- | --- | --- |
|  | **FIN** | **FIN** |
| ESTCON | **-0.013***** | **-0.014***** |
|  | **(-2.742)** | **(-2.984)** |
| SIZE |  | 0.003 |
|  |  | (1.177) |
| LEV |  | -0.004 |
|  |  | (-0.274) |
| ROA |  | -0.092*** |
|  |  | (-2.976) |
| FIX |  | -0.119*** |
|  |  | (-8.581) |
| PAY |  | -0.466 |
|  |  | (-0.139) |
| BSIZE |  | -0.016 |
|  |  | (-1.530) |
| DUAL |  | 0.010*** |
|  |  | (2.677) |
| TOP2_10 |  | -0.001*** |
|  |  | (-9.003) |
| MARKET |  | 0.001 |
|  |  | (0.819) |
| AGDP |  | 0.174** |
|  |  | (2.151) |
| AGDP^2^ |  | -0.008** |
|  |  | (-2.233) |
| YEAR | YES | YES |
| IND | YES | YES |
| _cons | 0.085*** | -0.839* |
|  | (4.311) | (-1.870) |
| N | 20514 | 20514 |
| Adj-R^2^ | 0.050 | 0.058 |
