# Supplementary material for: Do local governments’ energy-saving target constraints inhibit financialization? Evidence from nonfinancial listed firms in China
Source: PLoS One. 2023 May 19;18(5):e0285342. doi: 10.1371/journal.pone.0285342 (PMC10198514; doi:10.1371/journal.pone.0285342)
Supplement: S3 Table — (DOCX) [file pone.0285342.s004.docx]

**S3 Table**. **Propensity score matching and entropy balancing**

| Panel A The results of the covariate balance test | | | | | | | | |
| --- | --- | --- | --- | --- | --- | --- | --- | --- |
| **Variable** | **Unmatched/**  **Matched** | **Treated** | | **Control** | **%bias** | | **T** | **P** |
| SIZE | U | 22.077 | | 22.204 | -10.6 | | -7.26 | 0 |
|  | M | 22.077 | | 22.104 | -2.2 | | -1.34 | 0.180 |
| LEV | U | 0.45677 | | 0.43146 | 12.7 | | 8.64 | 0 |
|  | M | 0.45677 | | 0.46225 | -2.9 | | -1.68 | 0.093 |
| ROA | U | 0.04207 | | 0.04271 | -1.3 | | -0.85 | 0.394 |
|  | M | 0.04207 | | 0.04119 | 1.7 | | 0.99 | 0.321 |
| FIX | U | 0.22835 | | 0.23199 | -2.2 | | -1.48 | 0.138 |
|  | M | 0.22835 | | 0.22528 | 1.8 | | 1.06 | 0.287 |
| PAY | U | 0.00061 | | 0.00064 | -4.5 | | -3.05 | 0.002 |
|  | M | 0.00061 | | 0.00064 | 1.3 | | 0.79 | 0.429 |
| BSIZE | U | 2.1769 | | 2.1386 | 19.6 | | 13.50 | 0 |
|  | M | 2.1769 | | 2.1746 | 1.2 | | 0.70 | 0.481 |
| DUAL | U | 0.80131 | | 0.74475 | 13.5 | | 9.06 | 0 |
|  | M | 0.80131 | | 0.80546 | -1 | | -0.61 | 0.544 |
| TOP2_10 | U | 20.651 | | 23.148 | -10.6 | | -7.26 | 0 |
|  | M | 20.651 | | 20.573 | -19.4 | | -13.21 | 0.719 |
| MARKET | U | 7.8751 | | 8.3659 | -25.3 | | -16.56 | 0 |
|  | M | 7.8751 | | 7.7954 | 4.1 | | 2.26 | 0.024 |
| AGDP | U | 11.193 | | 11.303 | -17.7 | | -12.38 | 0 |
|  | M | 11.193 | | 11.201 | -1.2 | | -0.68 | 0.494 |
| AGDP^2^ | U | 125.72 | | 128.09 | -17.2 | | -12.03 | 0 |
|  | M | 125.72 | | 125.86 | -1.0 | | -0.57 | 0.570 |
| Panel B The results of propensity score matching method | | | | | | | | |
|  | | | **(1)** | | | **(2)** | | |
|  | | | **FIN** | | | **FIN** | | |
| ESTCON | | | **-0.019***** | | | **-0.020***** | | |
|  | | | **(-2.914)** | | | **(-2.845)** | | |
| SIZE | | |  | | | 0.003 | | |
|  | | |  | | | (0.793) | | |
| LEV | | |  | | | -0.001 | | |
|  | | |  | | | (-0.065) | | |
| ROA | | |  | | | -0.099* | | |
|  | | |  | | | (-1.896) | | |
| FIX | | |  | | | -0.144*** | | |
|  | | |  | | | (-6.538) | | |
| PAY | | |  | | | -6.174 | | |
|  | | |  | | | (-1.132) | | |
| BSIZE | | |  | | | -0.020 | | |
|  | | |  | | | (-1.236) | | |
| DUAL | | |  | | | 0.002 | | |
|  | | |  | | | (0.240) | | |
| TOP2_10 | | |  | | | -0.001*** | | |
|  | | |  | | | (-6.801) | | |
| MARKET | | |  | | | 0.000 | | |
|  | | |  | | | (0.104) | | |
| AGDP | | |  | | | 0.186* | | |
|  | | |  | | | (1.722) | | |
| AGDP^2^ | | |  | | | -0.009* | | |
|  | | |  | | | (-1.784) | | |
| YEAR | | | YES | | | YES | | |
| IND | | | YES | | | YES | | |
| _cons | | | 0.104*** | | | -0.852 | | |
|  | | | (3.903) | | | (-1.413) | | |
| N | | | 10083 | | | 10083 | | |
| Adj-R^2^ | | | 0.043 | | | 0.052 | | |
| Panel C The results of entropy balancing method | | | | | | | | |
|  | | | **(1)** | | | **(2)** | | |
|  | | | **FIN** | | | **FIN** | | |
| ESTCON | | | **-0.015***** | | | **-0.015***** | | |
|  | | | **(-3.366)** | | | **(-3.173)** | | |
| SIZE | | |  | | | 0.003 | | |
|  | | |  | | | (0.894) | | |
| LEV | | |  | | | -0.015 | | |
|  | | |  | | | (-0.936) | | |
| ROA | | |  | | | -0.112*** | | |
|  | | |  | | | (-2.731) | | |
| FIX | | |  | | | -0.129*** | | |
|  | | |  | | | (-7.533) | | |
| PAY | | |  | | | -3.266 | | |
|  | | |  | | | (-0.756) | | |
| BSIZE | | |  | | | -0.015 | | |
|  | | |  | | | (-1.179) | | |
| DUAL | | |  | | | 0.011** | | |
|  | | |  | | | (2.191) | | |
| TOP2_10 | | |  | | | -0.001*** | | |
|  | | |  | | | (-7.794) | | |
| MARKET | | |  | | | 0.002 | | |
|  | | |  | | | (1.197) | | |
| AGDP | | |  | | | 0.211** | | |
|  | | |  | | | (2.471) | | |
| AGDP^2^ | | |  | | | -0.010** | | |
|  | | |  | | | (-2.558) | | |
| YEAR | | | YES | | | YES | | |
| IND | | | YES | | | YES | | |
| _cons | | | 0.086*** | | | -1.028** | | |
|  | | | (3.954) | | | (-2.171) | | |
| N | | | 21578 | | | 21578 | | |
| Adj-R^2^ | | | 0.046 | | | 0.054 | | |
