# Supplementary material for: Do local governments’ energy-saving target constraints inhibit financialization? Evidence from nonfinancial listed firms in China
Source: PLoS One. 2023 May 19;18(5):e0285342. doi: 10.1371/journal.pone.0285342 (PMC10198514; doi:10.1371/journal.pone.0285342)
Supplement: S4 Table — (DOCX) [file pone.0285342.s005.docx]

**S4 Table**. **Energy-saving target constraints intensity analysis**

|  | **(1)** | **(2)** |
| --- | --- | --- |
|  | **FIN** | **FIN** |
|  | **High Constraint** | **Low Constraint** |
| ESTCON | **-9.686**** | **4.392** |
|  | **(-2.205)** | **(1.620)** |
| SIZE | -0.006 | 0.002 |
|  | (-0.392) | (0.406) |
| LEV | -0.064 | 0.014 |
|  | (-0.892) | (0.529) |
| ROA | -0.234 | -0.057 |
|  | (-1.127) | (-0.784) |
| FIX | -0.212*** | -0.116*** |
|  | (-3.008) | (-3.921) |
| PAY | -32.615 | -0.825 |
|  | (-1.515) | (-0.113) |
| BSIZE | -0.067 | -0.006 |
|  | (-1.036) | (-0.318) |
| DUAL | 0.013 | 0.005 |
|  | (0.441) | (0.516) |
| TOP2_10 | -0.001 | -0.002*** |
|  | (-1.363) | (-5.486) |
| MARKET | 0.005 | 0.000 |
|  | (0.551) | (0.100) |
| AGDP | 0.839** | 0.198 |
|  | (2.327) | (1.392) |
| AGDP^2^ | -0.038** | -0.009 |
|  | (-2.239) | (-1.486) |
| YEAR | YES | YES |
| IND | YES | YES |
| _cons | -4.157** | -0.916 |
|  | (-2.110) | (-1.158) |
| N | 1183 | 5551 |
| Adj-R^2^ | 0.040 | 0.041 |
